# Sex-dependent gene co-expression in the human body

**DOI:** 10.1038/s41598-021-98059-9

**Published:** 2021-09-21

**Authors:** Robin J. G. Hartman, Michal Mokry, Gerard Pasterkamp, Hester M. den Ruijter

**Affiliations:** 1 Laboratory of Experimental Cardiology, University Medical Center Utrecht, University Utrecht, Heidelberglaan 100, 3584CX Utrecht, The Netherlands; 2 Central Diagnostics Laboratory, University Medical Center Utrecht, University Utrecht, Utrecht, The Netherlands

**Keywords:** Gene regulatory networks, Regulatory networks, Systems analysis, Anatomy, Medical research, Molecular medicine

## Abstract

Many pathophysiological mechanisms in human health and disease are dependent on sex. Systems biology approaches are successfully used to decipher human disease etiology, yet the effect of sex on gene network biology is mostly unknown. To address this, we used RNA-sequencing data of over 700 individuals spanning 24 tissues from the Genotype-Tissue Expression project to generate a whole-body gene co-expression map and quantified the sex differences per tissue. We found that of the 13,787 genes analyzed in 24 tissues, 29.5% of the gene co-expression is influenced by sex. For example, skeletal muscle was predominantly enriched with genes co-expressed stronger in males, whereas thyroid primarily contained genes co-expressed stronger in females. This was accompanied by consistent sex differences in pathway enrichment, including hypoxia, epithelial-to-mesenchymal transition, and inflammation over the human body. Furthermore, multi-organ analyses revealed consistent sex-dependent gene co-expression over numerous tissues which was accompanied by enrichment of transcription factor binding motifs in the promoters of these genes. Finally, we show that many sex-biased genes are associated with sex-biased diseases, such as autoimmunity and cancer, and more often the target of FDA-approved drugs than non-sexbiased genes. Our study suggests that sex affects biological gene networks by differences in gene co-expression and that attention to the effect of sex on biological responses to medical drugs is warranted.

## Introduction

Sex influences the processes underlying development and disease. Sex differences in the prevalence of diseases are appreciated today, e.g. autoimmune diseases are more common in females, whereas non-reproductive cancers are more common in males^1^. At the basis of this diversity in disease prevalence are the molecular and genetic differences between male and female cells driven by gonadal hormones and sex chromosomes^2^. Previous efforts have shown that the human transcriptome shows sex-differential gene expression over different tissues^3,4^.

Systems and network biology are successfully used to find disease targets based on the complexity of biological systems, and recent studies have shown that sex affects biological gene networks^5,6^. We have previously shown that sex-stratified gene regulatory networks can find sex-specific key drivers of disease^7^. However, a whole-body map of sex differences in gene co-expression is lacking, while gene co-expression underlies biological gene networks. We hypothesize that substantial and overarching sex differences exist in gene network activity as measured by gene co-expression in tissues of the human body.

Therefore, we created a whole-body map of sex differences in gene co-expression, based on gene co-expression and expand on the overarching sex-biased pathways. Next, we analyzed gene co-expression patterns and their regulation by sex down to the tissue level. Lastly, we determined whether sex-biased genes are important for sex-biased disease, and whether or not they are targets of FDA-approved drugs.

## Materials and methods

### Study population

**Figure 1:**
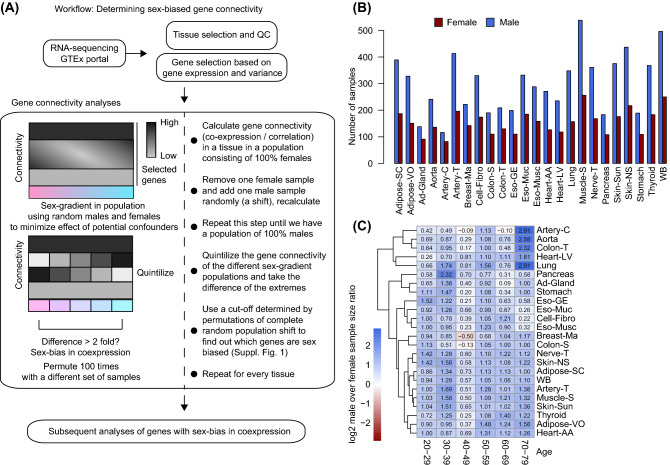
Workflow of sex-stratified gene co-expression analyses. (**A**) The workflow of analyses performed in this study are shown. We started by analysing publicly available RNA-sequencing data from the GTEx Portal. We selected tissues with a RIN > 6.0, and with at least 80 samples of both sexes. Subsequently, we determined sex-biased gene co-expression in those 24 tissues that met our criteria as elaborated upon in the large box. See Suppl. Fig. 1A. for an in depth workflow. (**B**) The amount of samples used over the 24 selected tissues is shown in a barplot. Red indicates female, blue indicates male. (**C**) Log_2_ sex-ratios (male over female) are shown in a heatmap. The rows show the tissues, while the columns indicate age decades.

The Genotype-Tissue Expression (GTEx) project RNA-sequencing data (v8)^8^ was used to describe a whole-body map of sex differences in gene co-expression. Analyses were performed as shown in Fig. 1 and Suppl. Fig. 1. To generate a whole-body map of sex differences in gene co-expression, we started our analysis with RNA-sequencing data from over 700 individuals in the GTEx project spanning more than 50 tissues. A comprehensive workflow of the analysis is shown in Fig. 1A (see Suppl. Fig. 1A for a more extensive flowchart). First, we selected samples that had at least a RIN > 6.0, a filter for the quality of RNA of the different samples. This cut-off is based on recommendations by the GTEx Portal. We performed principal component analyses on all tissues passing our criteria (Suppl. Fig. 2).

### Bioinformatics

Transcripts-per-million (TPM) expression data were downloaded from the GTEx Portal (v8), along with associated sample annotations and subject phenotypes. All analyses were performed in R (v4.0.4) within RStudio (v1.1.463). Principal component analyses and plots were done with 1000 genes that were most variable in expression for each tissue as defined by expression variance. Code for all analyses can be found at https://github.com/rjghartman.

### Co-expression analyses

Genes for the whole-body map of gene co-expression were selected on the basis of variance and expression. A log_2_ expression cut-off of 1 TPM on average for each gene and variance of at least 1 were used to ensure enough information for co-expression analyses. Gene co-expression values were calculated using log_2_ TPM values for selected genes using WGCNA^9^ (v1.70-3) with the exponent equal to five. Repeated co-expression analyses using different populations with regards to sex-ratio are shown in Suppl. Fig. 1. Sex bias determination for individual genes is described in Suppl. Fig. 1. In short, gene co-expression analyses were calculated in a tissue-specific manner. The co-expression values were first calculated in a population consisting of 100% random female samples, and then repeated for a population with one random female sample removed, while adding one random male sample. This was repeated until the population consisted of 100% male samples. Next, in order to find genes that showed a sex-bias, we quintilized the calculated co-expression values over the population shift and took the difference between the extremes (i.e. between populations consisting mostly of female samples and populations consisting of mostly male samples). The cut-off for sex-bias was determined by permuting co-expression shifts 100 times using random samples in each population, instead of a gradient by sex (Suppl. Fig. 1A, box 3). We calculated log-fold changes of co-expression differences of 100 permutations in 3 different tissues; artery-C, heart-AA, and ad-Gland (Suppl. Fig. 1B). The amount of genes passing an absolute cut-off of log2-fold change over 1 was always higher in co-expression shifts with a sex-gradient as compared to co-expression shifts with random samples, indicating that sex is an important determinant in co-expression (Suppl. Fig. 1C). Genes were called sex-biased if the log2-fold change between the extreme quintiles was higher than 1. Lastly, we repeated this entire procedure 100 times, with different random samples every permutation. In the end, we only continued with sex-biased genes that were consistently called sex-biased in at least 20 permutations (Suppl. Fig. 3C).

A similar analysis was performed to determine the effect of menopause on co-expression in females. Since the average age of menopause is 51 in women in the USA, we created a dummy variable for females > 50 years of age and < 50 years of age. Next, the co-expression analyses were performed shifting over this dummy variable. To determine whether a gene was affected by menopause, we used the same cut-off and compared once whether a sex-biased gene was also a menopause-affected gene.

Differential gene expression analysis between the sexes was performed using DESeq2 (v1.30.1) on all selected samples on the rounded TPM data. Genes were called significantly differentially expressed if FDR < 0.1. Differential expressed genes higher expressed in females samples were compared to female-biased genes regarding co-expression, and genes higher expressed in males were compared with male-biased genes regarding co-expression, to determine the overlap between differential gene expression and bias in co-expression.

### Enrichment analyses

**Figure 2:**
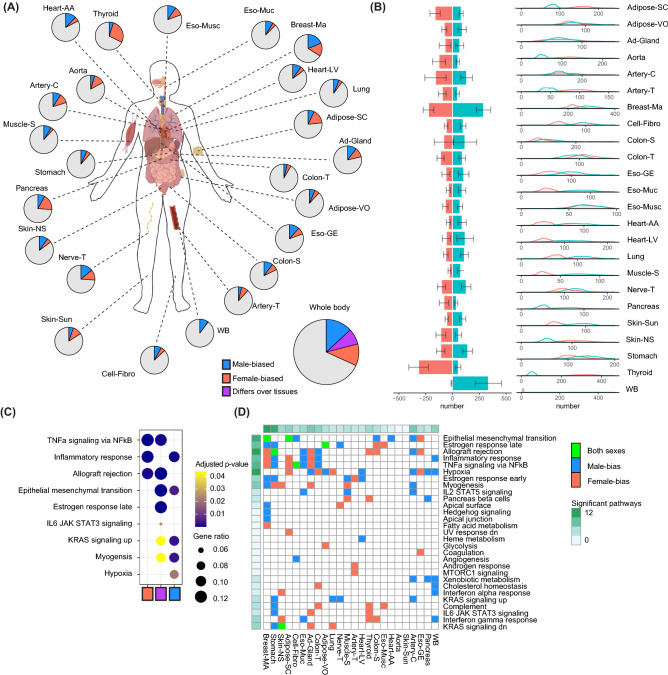
A whole-body map of sex differences in gene co-expression. (**A**) A cartoon of the human body is shown with the tissues that are used in our study. For each tissue, the number of genes that are either male- (blue) or female-biased (red) are shown in a piechart. The bigger piechart shows all genes combined over all tissues, with purple indicating genes that differ in bias over multiple tissues. (**B**) The exact number of genes that are either male- or female-biased per tissue is shown in a barplot with a 95% confidence interval as generated by permutation analysis. Adjacent density plots show the distribution of number of sex-biased genes over the different permutations. (**C**) A dotplot is shown for the top hallmark enrichments for the whole-body genes that are female-, male- or either sex-biased. Color indicates *p-*value, while size of the dot indicates gene ratio. (**D**) A heatmap is shown that indicates significantly enriched hallmarks over the different tissue. The color within the heatmap indicates whether the enrichment is significant in female-biased genes (red), male-biased genes (blue) or both (green) for that tissue-hallmark combination. Significant pathways indicates the total number of significant enrichments for the different tissues and the different hallmarks.

Hallmark enrichments were used to get an overview of well-curated lists of common pathways^10^. We calculated gene enrichment of genes with sex-biased gene co-expression with the clusterProfiler^11^ package (v3.18.1) by using the compareCluster function on different subsets (i.e. tissue-specific, male-specific, female-specific). An enrichment was deemed significant if p < 0.05. Only significant enrichments are shown in Fig. 2D. The enrichment heatmap was plotted using the “pheatmap” package (v1.0.12) in R. Enrichment for cell marker identity was performed using the PanglaoDB^12^ augmented 2021 dataset via Enrichr^13^, which was subsequently analysed with clusterProfiler. Enrichment map plots were visualized using the emapplot function in the enrichplot package. To determine enrichment for sex-biased diseases, such as autoimmunity and non-reproductive cancers, we matched terms in the DisGeNET^14^ dataset, also downloaded via Enrichr.

### Motif analyses

Motif enrichment analysis was performed using RcisTarget^15^ (v1.10.1). In short, RcisTarget identified transcription factor binding motifs that were enriched in our lists of sex-biased genes. Motifs were subset to only the Hocomoco database. We set the cut-off for the normalized enrichment score (NES) to 0 to calculate NES for the majority of the motifs (instead of only the significant ones), after which we only took the ones calculated in both the female- and the male-biased genes (406 motifs). The motif rankings version used was human hg38 refseq-r80 for 500 bp upstream and 100 bp downstream the transcription start site. The motif-to-TF annotation version used was human v9.

### Gene-disease associations

To generate a list of genes associated with disease, we used the DisGeNET database^14^. We filtered the dataset on diseases for which at least 100 genes were annotated, and subsetted the gene-disease association based on the higest evidence score by removing any associations that were below the 90th% quantile. These genes were subsequently compared with sex-biased genes and FDA-approved drug targets, as discussed below. The venn-diagram was made using the vennDiagram package in R (v1.6.20).

### FDA-approved drugs associations

FDA-approved drug targets were obtained from a comprehensive map of FDA drugs and their targets^16^. To determine whether the overlap between sex-biased disease genes and FDA-approved drug targets was more prominent than non sex-biased genes, we permuted the overlap between non sex-biased genes and FDA-approved drug targets 10,000 times. The p-value for permutation was determined as the proportion of overlaps that was lower than the overlap between sex-biased disease genes and FDA-approved drug targets. The same logic was applied to overlap with disease genes. The list used for FDA-approved drug targets can be found in the Supplemental Dataset.

## Results

### The workflow of the study

The workflow of the study is depicted in Fig. 1A. The majority of samples within the GTEx Portal were male (Fig. 1B). Since we needed enough power to calculate sex differences in gene co-expression, we assessed tissues with at least 80 samples of both sexes. This reduced the number of tissues to 24, which were used in all analyses. We found that male and female samples were clustering mostly separately in breast-MA tissue, indicating that the transcriptome of male and female cells is most variable in breast-MA tissue. As the life expectancy of females is higher than males^17^, we determined the age distribution in male and female tissues. No major differences were found across the different life-decades in the tissues (Fig. 1C). Almost all the different age-tissue combinations were male-biased in sample size ranging from 1.06 times as many males in the adrenal gland (60–69 years of age) to 7.5 times as many males in the lung (70–79 years of age), indicating that the age distribution over all tissues is similar. One major exception was mammary breast tissue (breast-MA) in 40–49 years of age, which is female-biased (0.7 times as many males).

### A whole body-map of sex differences in gene co-expression

We found sex-dependent gene co-expression for 4062 genes out of 13,787 (29.5%) genes tested over all tissues combined (Fig. 2A). The total number of genes that are exclusively female-biased is 1378, while 1680 are exclusively male-biased. To generate a whole body-map of sex differences in gene co-expression, we calculated co-expression of highly variable and expressed genes in populations that differ in sex-ratio from 100% female to 100% male for every tissue (Suppl. Fig. 1A, box 1). We started by calculating the connectivities in females. Subsequently, we removed one female sample and added one male sample and recalculated the co-expression. These concurrent steps always were randomized in their samples, hence, the effect of age, and any other potential confounders is negligible. From left to right on the x-axis of the heatmap in Fig. 1A, females were subsequently replaced by males until the population resulted in 100% males. Then, quintiles of these populations varied by sex were formed by taking median co-expression values (Suppl. Fig. 3A). The amount of sex-bias in co-expression was subsequently calculated by dividing the co-expression value for the 5th quintile (containing the majority of male populations) by the 1st quintiles (containing the majority of female populations). We repeated this entire procedure 100 times for each tissue, having random samples again every permutation. The amount of genes called sex-biased in every iteration was stable (Fig. 2B). Of the 4062 genes, 1004 show sex-bias toward both sexes, but differed per tissue. The tissue that presented with most sex differences in gene co-expression is Breast-MA, with 380 genes male-biased and 306 genes female-biased. Tissues that presented mostly with male-based co-expression were whole blood (WB) and skeletal muscle (muscle-S), while thyroid and subcutaneous adipose tissue (adipose-SC) presented mostly with female-based co-expression (Fig. 2A,B). Interestingly, fibroblasts, and thus cells per se, also presented with sex-bias in co-expression, indicating that tissue composition did not fully explain sex differences in gene co-expression. A complete list of genes that present with either male- or female-bias in gene co-expression for each tissue and their chromosomal location, as well as how often out of the 100 iterations they were called sex-biased can be found in the Supplemental Dataset. Since the age of the subjects within GTEx ranges from the decades 20–29 till 70–79 and menopause is a large driver of physiological differences within females, we determined the effects of menopause within females on the sex-biased gene co-expression. We performed a similar analyses grouping females below the age of 50 and above the age of 50, and determined the differences in co-expression driven by menopause. Of all sex-biased genes, an average of 30–32% was potentially affected by menopause (Suppl. Fig. 4A). Furthermore, to determine whether genes that show a sex-bias in co-expression are not necessarily differentially expressed between the sexes, we performed sex-differential gene expression analysis in all tissues. Overlap between sex-differential gene expression and sex-bias in co-expression was low (Suppl. Fig. 4B), being the highest in breast-MA tissue for both sexes.

### Consistent sex-dependent gene co-expression over pathways

Gene hallmark analysis showed that the entirety of female-biased genes over all tissues were enriched for immune response hallmarks, such as inflammatory response and allograft rejection (Fig. 2C). All male-biased genes together in all tissues were enriched for myogenesis, and epithelial to mesenchymal transition. The 1004 genes that presented with either a male- or a female-bias over differing tissues were enriched for estrogen responses and inflammation pathways (Fig. 2C). To determine sex-dependent pathway activity over the different tissues separately, we calculated gene enrichments using hallmarks in female- and male-biased genes per tissue (Fig. 2D). Some hallmarks are more commonly significantly enriched in either male- or female-biased (or both) over different pathways than others (the rows in Fig. 2D), e.g. epithelial-to-mesenchymal transition and inflammatory response show up in multiple tissues, whereas enrichment for androgen resposne is only found for male-biased genes in Artery-T. The amount of significant hallmarks also differ per tissue (the columns in Fig. 2D), e.g. stomach has multiple significant hallmarks for male-biased genes, whereas aorta contains none. Inflammatory hallmarks show consistent differences over different tissues, such as TNF-a signalling and inflammatory responses, that are significantly enriched in female-biased genes in both Breast-Ma and Adipose-SC.

### Multi-organ sex differences and regulation

**Figure 3:**
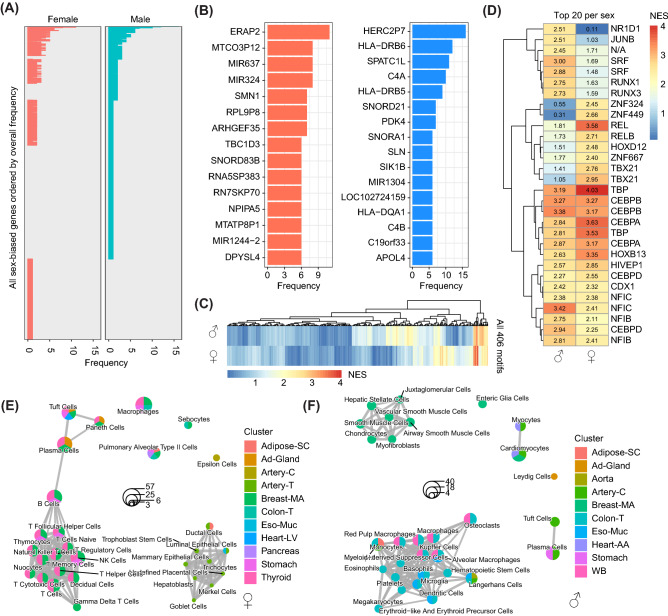
Tissue-transcending sex differences and regulation. (**A**) A barplot indicates how often sex-biased genes are consistent over multiple tissues (female on the left, male on the right). No bar for one sex means that that gene is exclusively sex-biased in the other sex. (**B**) A barplot shows the sex-biased genes that are sex-biased consistently in at least 6 tissues consistently, with ERAP2 being the top gene for females, and HERC2P7 being the top hit for males. (**C**) A heatmap shows the normalized enrichment score (NES) of 406 tested motifs for male-biased (top) and female-biased (bottom) genes in at least two tissues. A NES > 3 is considered significantly enriched. (**D**) A heatmap shows the NES for the top 20 male- and female-biased motifs. (**E**) A cell identity marker enrichment map is plotted for female-biased genes. The top 30 enrichments are shown, with color indicating for which tissue the enrichment was found, while size indicates the overlapping gene count. (**F**) A cell identity marker enrichment map is plotted for male-biased genes. The top 30 enrichments are shown, with color indicating for which tissue the enrichment was found, while size indicates the overlapping gene count.

As tissues in our body are connected by endocrine and paracrine mechanisms, we analysed gene co-expression patterns and their regulation by sex over multiple tissues together. For female-biased genes, 28.2% was female-biased in more than one tissue, while 24.9% of male-biased genes were male-biased in more than one tissue (Fig. 3A). The majority of these genes were consistently male- or female-biased in two tissues, as compared to more three and higher. An extreme example is the gene *ERAP2* which was female-biased in 11 tissues, while the gene that was most consistently male-biased (in 16 tissues) was *HERC2P7* (Fig. 3B). We also removed genes that were male- or female-biased in different tissues to have a better understanding of genes specifically co-expressed in one sex only. This led to 20.0% of all exclusively female-biased genes being female-biased in more than one tissue, while this number was 16.2% for male-bias. The most common exclusively male-biased genes were *STC2, ANGPTL4, TPSAB1, TPSB2, SNORA33, SNORD* being consistently male-biased in five tissues. The most common exclusively female-biased genes were *APOC1, IGKJ3, USP32P2, and HLA-DOB*, being consistently female-biased in five tissues. As differences in overall gene regulation may underlie consistent patterns of gene co-expression, we performed motif analysis on male- and female-biased genes. Normalized enrichment scores (NES) for 406 tested motifs differed between male- and female-biased genes, as visible from the differences in clustering (Fig. 3C). The top 20 motifs for both male-biased and female-biased genes are shown in Fig. 3D. Some potential transcription factors (TFs) driving higher gene co-expression for male-biased genes are *SRF* and *RUNX1/RUNX3*. Some potential TFs for female-biased genes are *TBX21*, and the inflammatory regulators *RELA/NFKB1*, underlining the inflammatory nature of the female-bias. Since differences in co-expression may also be driven by tissue and sex differences in cell composition, we analysed whether sex-biased genes in different tissues were enriched for markers of cell identity (Fig. 3E). Female-biased genes of breast-MA and thyroid tissue were strongly enriched for markers of multiple immune cell-types, whereas male-biased genes of breast-MA were enriched for markers of mesenchymal cells (Fig. 3F). Furthermore, whole blood, colon-T and eso-Muc were showing strong signs of immune cell-types for genes with stronger co-expression in males.

### Importance of sex-dependent gene co-expression for diseases and drug discovery

**Figure 4:**
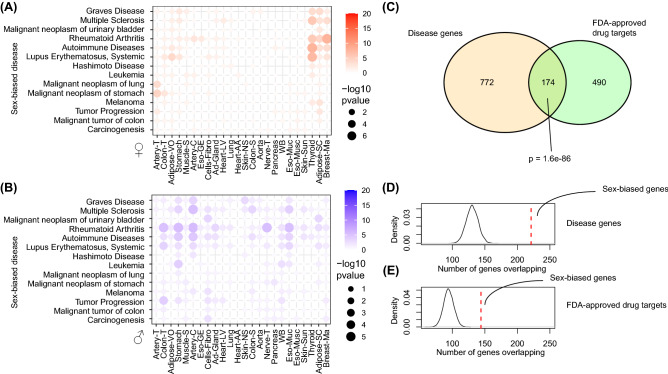
Importance of sex-biased co-expression exemplified by disease and FDA-approved drugs. (**A**) A heatmap shows the enrichment for female-biased genes over sex-biased disease such as autoimmune disease and non-reproductive cancer. Disease are shown in the rows, while the tissues are placed on the columns. Color and size of dot indicate –log10 p-value. (**B**) A heatmap shows the enrichment for male-biased genes over sex-biased disease such as autoimmune disease and non-reproductive cancer. Disease are shown in the rows, while the tissues are placed on the columns. Color and size of dot indicate – log10 p-value. (**C**) A venn-diagram highlights the overlap between disease-associated genes and gene targets of FDA-approved drugs. (**D**) A density-plot of amount of genes overlapping shows the permuted distribution of random genes and their overlap with disease genes. The vertical line highlights the observed value for sex-biased genes. (**E**) A density-plot of amount of genes overlapping shows the permuted distribution of random genes and their overlap with FDA-approved drug target genes. The vertical line highlights the observed value for sex-biased genes.

Systems biology provides an important step in the discovery of disease targets. To test the clinical importance of sex within systems biology, we determined whether sex-biased genes are associated with sex-biased disease, such as autoimmunity and non-reproductive cancers^1^ (Fig. 4A). Strikingly, we found a strong cluster of female-biased genes enriched for female-biased autoimmune diseases such as systemic lupus erythematosus and multiple sclerosis (Fig. 4A). On top of that, female-biased genes in thyroid were strongly associated with Graves’ disease, an autoimmune disease of the thyroid more prevalent in females^1^. Enrichments for non-reproductive cancers were not as significant for female-biased genes, while male-biased genes in different tissues were more often enriched for carcinogenesis and tumor progression (Fig. 4B). Next, we wanted to determine whether sex-biased genes were enriched for targeting by FDA-approved drugs. First, we calculated the overlap between genes associated with disease and FDA-approved drugs, which highlighted the expected association between disease genes and genes targeted by FDA-approved drugs (p = 1.6e−86, Fig. 4C). Second, we determined whether sex-biased genes, as opposed to genes that were not sex-biased, were more often found among disease genes. We found significant enrichment of genes that show a sex-bias in their co-expression among genes that are important for disease. The overlap was larger for sex-biased genes than for genes that showed no sex bias (permutation *p*-value < 0.0001, Fig. 4D). Lastly, we wanted to see whether there was enrichment of sex-biased genes among genes targeted by FDA-approved drugs. This association was much stronger for sex-biased genes than for genes that did not show a sex-bias (permutation *p*-value < 0.0001, Fig. 4E). A complete list of FDA-drug target associated sex-bias genes is provided in the Data Supplement. Our findings highlight the importance of incorporating sex in systems biology for improved understanding of diseases and the search for novel drug targets.

## Discussion

We generated a whole-body map of sex differences in gene co-expression and highlight that sex is an important factor in systems biology: Overall, gene co-expression is sex-biased for 29.5% of the 13,787 genes analysed. As gene co-expression is the basis for further construction of regulatory gene networks to detect novel and druggable targets for disease, biological networks are more applicable to both women and men when analysed in a sex-stratified way. Interestingly, we found consistent sex differences in gene enrichment of hallmarks present over multiple tissues in the human body. Furthermore, we showed that gene co-expression varied by sex over multiple tissues, which was also accentuated by our analyses on regulatory DNA motifs. Lastly, we highlighted the importance of sex differences in gene co-expression for diseases, target selection and drug discovery.

Sex-dependent gene co-expression might be driven by sex differences in (a combination of) sex chromosomes, gonadal hormones, and gender differences. The X- and Y-chromosomes harbor global gene regulators that are present in different doses between the sexes^18^. These genes can regulate the expression of genes located on the autosomes^19^, leading to differences in gene expression and co-expression between the sexes, and ultimately gene regulatory networks. Indeed, a recent report on sex differences in gene expression and gene regulatory networks in the GTEx portal has shown large differences between the sexes in gene regulatory networks^20^. Our study differs with regards to those methods, since we use co-expression values as a basis for systems biology, as opposed to transcription factor driven networks. However, both studies agree that sex is an important factor to take into account in the sex-specific genetic architecture of gene regulatory networks that govern health and disease. Furthermore, a study by our own group has shown that sex-stratified gene regulatory networks illuminate sex-specific disease biology and point to sex-specific key drivers of disease as well^7^.

Gonadal hormones, such as estrogens and androgens, affect gene expression via multiple mechanisms. They may bind nuclear receptors, which subsequently translocate to the nucleus to bind promoter sequences, inducing or repressing transcription. Other mechanisms on transcription are less direct, such as interactions with epigenetic modifiers or signalling via G-protein coupled receptors. As the gonadal hormone arsenal varies immensely between males and females, gonadal hormones are strong candidates for influencing the sex-specific landscape of pathway activity. Importantly, we found estrogen responses to be enriched in genes that present with sex-bias in co-expression (Fig. 2C,D). Furthermore, on average, ~ 30–32% of genes with a sex-bias in co-expression are potentially affected by menopause (Suppl. Fig. 4A).

We found clear sex-dependent gene co-expression in general pathways over the human body. For example, pathways that were inflammatory in nature, such as TNF-a signalling and allograft rejection, were more commonly female-biased over multiple healthy tissues. This was also underlined by stronger enrichment for inflammatory and immune-related regulatory motifs in the promoters of these genes. Sex differences in the immune system have been thoroughly described^1^, and our data echoes the importance of sex in immunity. The tissue with the most female-biased (inflammatory) pathways was thyroid. Perhaps not coincidentally, autoimmune disease of the thyroid, such as Hashimoto’s thyroiditis and Graves’ disease, are more prevalent in females as compared to males^1^. Interestingly, female-biased genes in thyroid were enriched for both Graves’ disease and Hashimoto disease as well (Fig. 4A).

Enrichment for pathways such as myogenesis, epithelial to mesenchymal transition and immune-related pathways may indicate differences in tissue composition between the sexes. We studied endothelial cell gene expression in GTEx as a marker for endothelial cell density (*VWF*, *CLDN5, CDH5 *“https://gtexportal.org/home/gene/CDH5”). The only tissue that showed a clear sex difference in endothelial cell expression was breast-MA. The strong sex difference in gene co-expression in breast-MA tissue may therefore also be driven by differences in cell composition, however, we cannot state this for other tissues, where endothelial cell gene expression did not differ as strongly. The compositional difference was underscored by a strong signal for immune cell markers in female-biased genes for breast-MA, and a coinciding signal for mesenchymal cell markers for the male-biased genes in breast-MA. One of the stronger TFBM for female-bias was *TBX21.* Mice lacking *TBX21* show increased visceral adiposity^21^, so an increased *TBX21* activity in females might help in explaining why females have less visceral adiposity as compared to males^22^. Tissue composition differences aside, we also found sex differences in gene co-expression and subsequent pathways in fibroblasts, highlighting that cells per se have different gene co-expression when comparing male to female cells which may contribute to sex differences in tissue composition.

Lastly, we found that genes with sex-biased gene co-expression are involved in sex-biased disease, as shown by overlap with gene-disease associations (Fig. 4A). Furthermore, we expected that disease-associated genes were enriched for targeting by FDA-approved drugs, which is underlined by our data. However, we now also found that sex-biased genes are more likely to be important for disease than genes that are not sex-biased, and on top of that, are also more likely to be FDA-approved drug targets. This warrants further sex-specific investigation into FDA-approved drugs for repurposing, as well as novel drugs, especially given the higher rate of adverse drug reactions in women.

### Limitations

An important message that needs to be reiterated is the underrepresentation of female samples in large databases, such as in our analysis (Fig. 1B). Unfortunately, this led us to exclude male samples from our analysis, to keep our studies equally powered for both sexes. We could not detect gender differences in our study, since the GTEx portal does not contain large questionnaires into behaviour and lifestyle choices of each individual. Larger epidemiological studies coupled to molecular deep phenotyping should allow for dissecting and understanding sex and gender influences simultaneously. Samples taken from individuals in GTEx are not specific for any disease and most likely represent healthy tissue. Sex-bias in specific disease settings might be different from those present in a healthy situation. Lastly, our analysis uses RNA-sequencing data, rendering us unable to look at protein levels and their modifications by sex, or at sex in the epigenetic landscape.

## Concluding remarks

The human whole-body map of sex differences in gene co-expression revealed major and consistent sex-dependency, in cells, single tissues, as well as over multiple tissues together. As gene co-expression is the basis for further construction of regulatory gene networks to detect novel and druggable targets for disease, biological networks should be analysed and interpreted in a sex-stratified way. This will accelerate drug development and ultimately benefit the health of both women and men.

## Supplementary Information

Supplementary Information 1.

Supplementary Information 2.

## Abbreviations

Adipose-SC: Subcutaneous adipose tissue
Adipose-VO: Adipose tissue from the visceral omentum
Ad-Gland: Adrenal gland
Artery-C: Coronary artery
Artery-T: Tibial artery
Breast-MA: Breast mammary tissue
Cell-Fibro: Fibroblasts
Colon-S: Colon sigmoid
Colon-T: Colon transverse
Eso-GE: Gastroesophageal junction
Eso-Muc: Esophagus mucosa
Eso-Musc: Esophagus muscularis
GTEx: Genotype-tissue expression
Heart-AA: Heart atrial appendage
Heart-LV: Heart left ventricle
Muscle-S: Skeletal muscle
Nerve-T: Tibial nerve
Skin-Sun: Skin (sun exposed)
Skin-NS: Skin (non-sun exposed, suprapubic)
TFBM: Transcription factor binding motif
WB: Whole blood
