# Supplementary material for: Sex-dependent gene co-expression in the human body
Source: Sci Rep. 2021 Sep 21;11:18758. doi: 10.1038/s41598-021-98059-9 (PMC8455523; doi:10.1038/s41598-021-98059-9)
Supplement: Supplementary file 1 — Supplementary Information 1. [file 41598_2021_98059_MOESM1_ESM.pdf]

**SUPPLEMENTAL FIGURES**

# Sex-dependent gene coexpression in the human body

Robin J.G. Hartman[1], PhD; Michal Mokry[1,2], MD PhD; Gerard Pasterkamp[2], MD PhD; Hester M. den Ruijter[1], PhD
[1] Laboratory of Experimental Cardiology, University Medical Center Utrecht, University Utrecht, Utrecht, The Netherlands;
[2] Central Diagnostics Laboratory, University Medical Center Utrecht, University Utrecht, Utrecht, The Netherlands

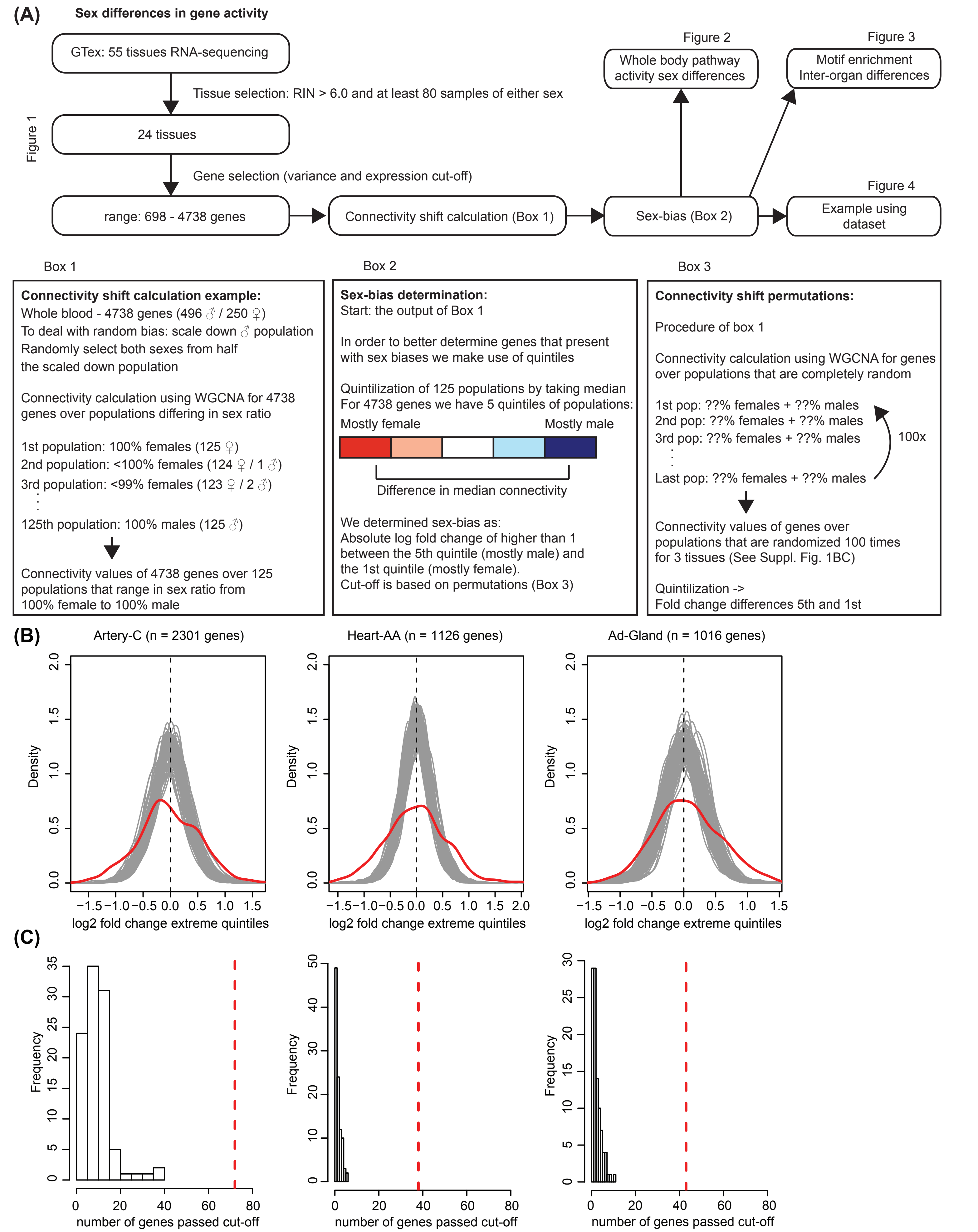


**Suppl. Fig. 1. In depth workflow analysis.** A) Workflow of the study and boxes to explain train of thought. B) Log fold change differences between the 5th and 1st quintile of the connectivity shifts are depicted for 100 permutations of random populations in artery-C, heart-AA, and ad-Gland (one grey = one permutation). The red line indicates the density distribution for the log fold changes of all genes in a sex-biased connectivity shift. C) Number of genes for the 100 permutatation cut-off log fold 1. The number of genes biased by a log fold 1 cut-off for the 100 permutations for 3 different tissues is shown in a histogram. The red dotted line indicates the number of genes with a log fold 1 cut-off in sex-biased connectivity analyses as used for the body map.

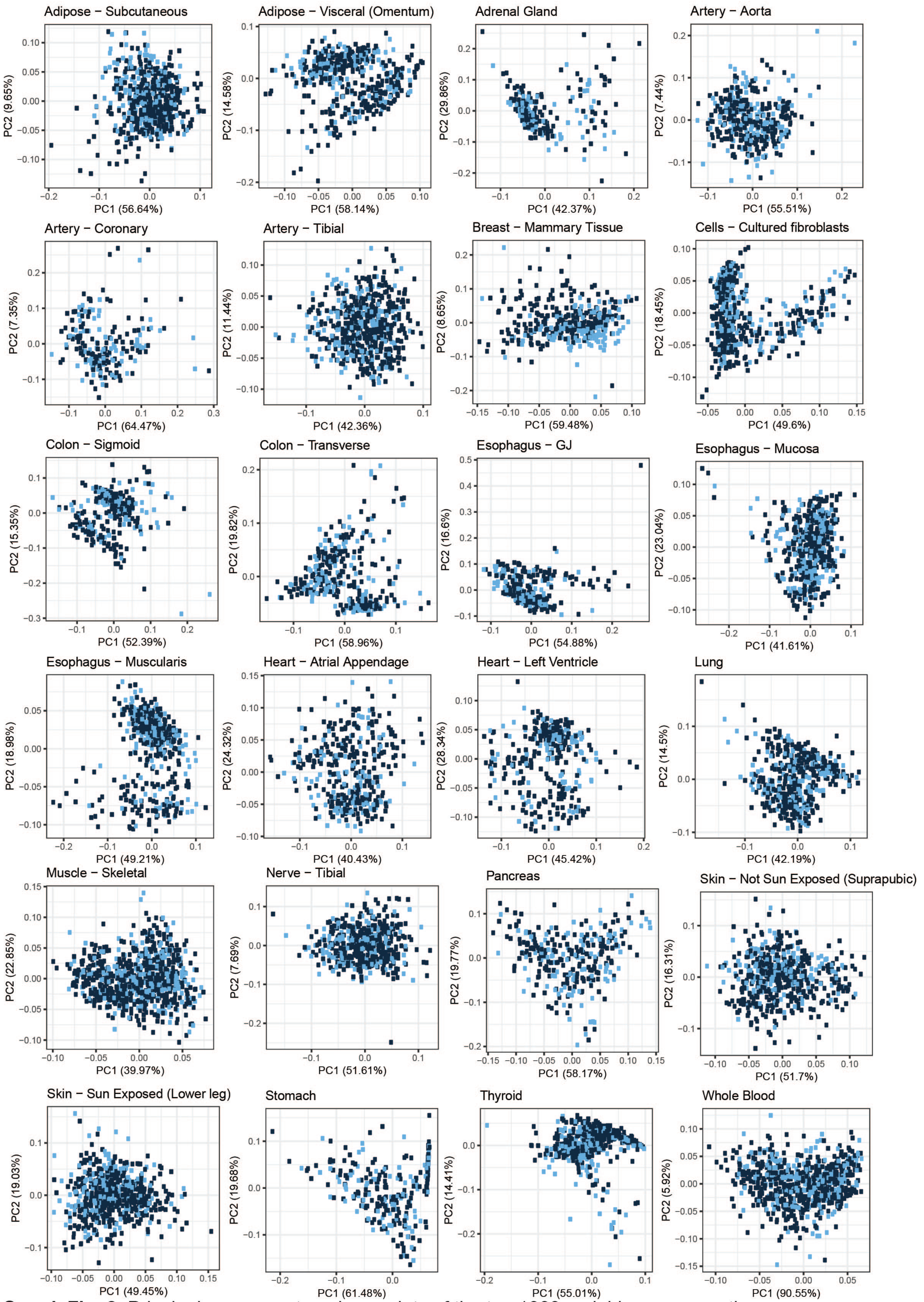


**Suppl. Fig. 2.** Principal component analyses plots of the top 1000 variable genes per tissue.
Lightblue = female, darkblue = male.

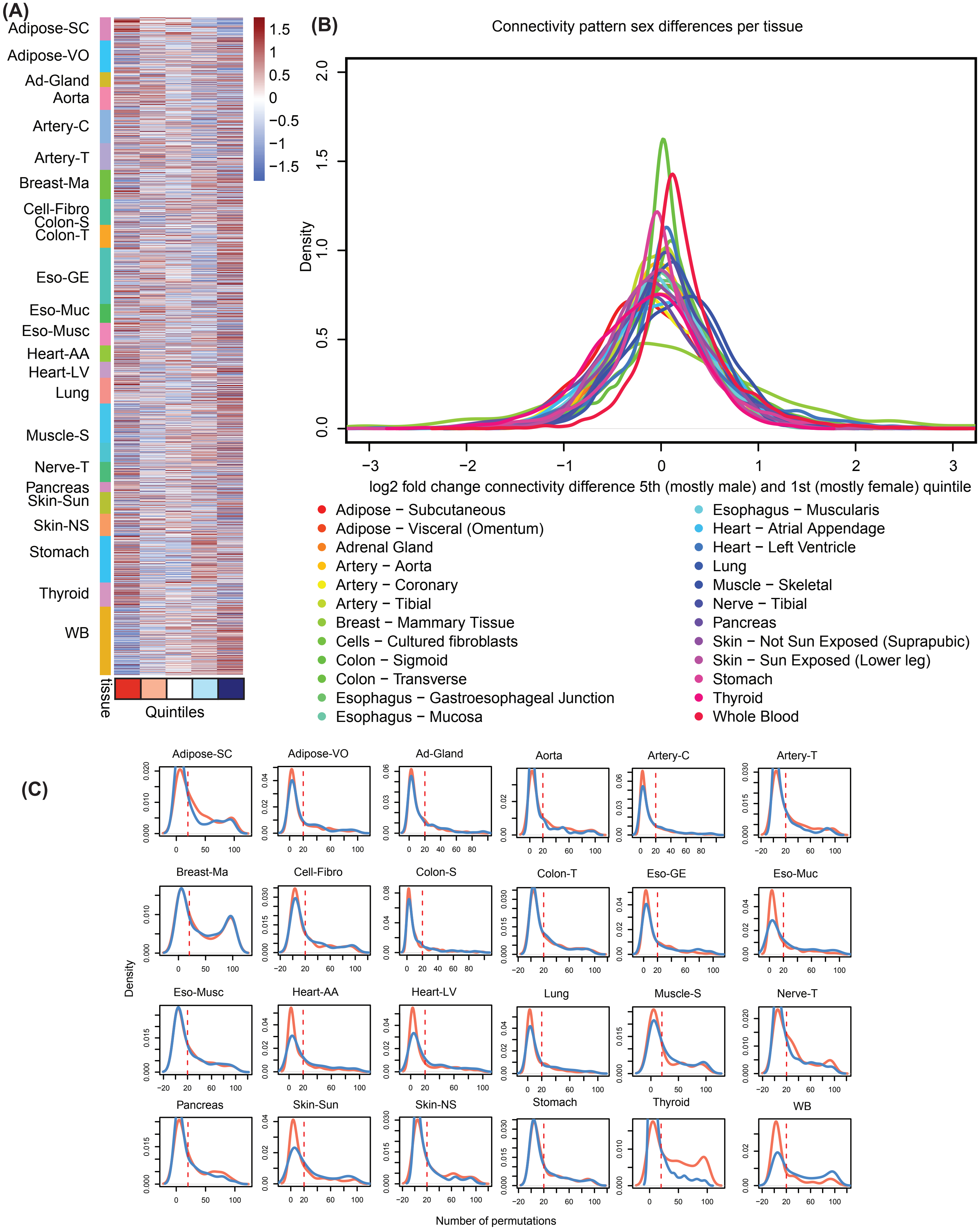


**Suppl. Fig. 3. Sex-bias in gene connectivity.** A) All connectivity values for all genes over all tissues tested are shown in a heatmap, divided in quintiles using their median. Quintile 1 = mostly female, quintile 5 = mostly male. B) Density plot for connectivity pattern differences. Log fold change differences between the 5th (mostly male) and 1st quintile (mostly female) of the connectivity shifts are depicted for all 24 tissues. Color indicates tissue. C) Density plots highlight the distribution of how often a gene is called sex-biased over the 100 different permutations.

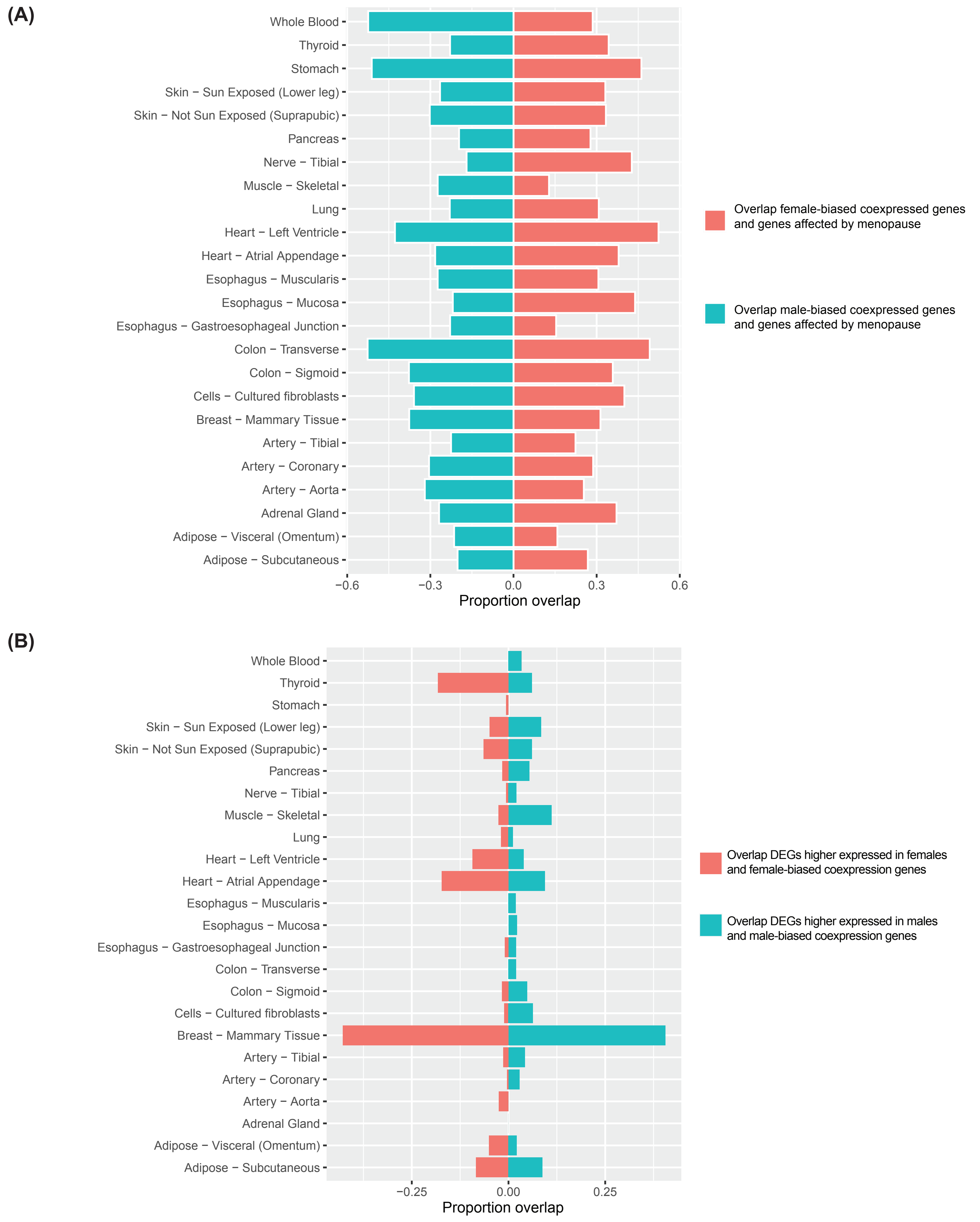


**Suppl. Fig. 4. Menopause and sex-biased coexpression, and differential gene expression.**

A). A barplot with the proportion of genes that is affected by menopause is shown. Red highlights the proportion of female-biased genes potentially affected by menopause, while blue highlights the proportion of male-biased genes affected by menopause. B). A barplot shows the overlap between sex-biased differential gene expression and sex-biased coexpression.
